# A study of the plant folk nomenclature of the Yi people in Xiaoliangshan, Yunnan Province, China, and the implications for protecting biodiversity

**DOI:** 10.1186/s13002-022-00504-0

**Published:** 2022-03-15

**Authors:** Yi-Won Addi, Yu Zhang, Xiao-Yong Ding, Chang-An Guo, Yu-Hua Wang

**Affiliations:** 1 Yunnan Key Laboratory for Wild Plant Resources, Kunming Institute of Botany, Chinese Academy of Sciences, 132# Lanhei Road, Heilongtan, Kunming, 650201 Yunnan China; 2 University of Chinese Academy of Sciences, Beijing, China

**Keywords:** Ethnobotany, Yi people, Xiaoliangshan, Indigenous botanical nomenclature

## Abstract

**Background:**

Folk plant nomenclature is a part of knowledge of indigenous people often used to distinguish plant species. This study aimed to document the folk botanical nomenclature of the Yi people in Xiaoliangshan, Yunnan Province, which has not been systematically investigated to date. The results of this study will assist in conserving biodiversity and the language of the Yi people and will promote the transmission of ethnobotanical knowledge.

**Methods:**

An ethnobotanical survey of plants used by the Yi people in Xiaoliangshan, Yunnan Province, was conducted from September 2019 to August 2021. Semi-structured interviews and key informant interviews were conducted to collect and document ethnobotanical information, which was then used to analyse the folk botanical nomenclature of the Yi people. In addition, the folk names of plants used by the Xiaoliangshan Yi community were compared with those of the Yi people living in the Daliangshan, where the environment is considerably different.

**Results:**

In this study, 266 informants were interviewed, and the names of 228 indigenous plants were extracted from 3088 use reports. The nomenclature used by the local Yi people is based on plant characteristics, plant habitat, plant use, and the local culture. By comparing the folk plant names of the Yi people in Xiaoliangshan with those of the Yi people in Daliangshan, we found that the plant names of the two places have some similarities and also with their own unique characters. The important folk plant names of the Yi people in Xiaoliangshan usually have a monosyllable non-binomial structure or have  and "divine attributes" in their names.

**Conclusions:**

The Yi people in Xiaoliangshan have a rich and diverse knowledge of plant naming determined by cultural, and environmental factors. The botanical nomenclature of the Yi people has distinct rules and characteristics, and the plant naming directly includes important plants that they believe to be used and protected, which is of great significance to the protection of biodiversity.

## Introduction

Plants have been studied and used throughout human history, and the vast number of botanical names in different languages attests to human plant knowledge [1]. Almost all cultures have names for indigenous plants [2], and as a unique naming system based on traditional ethnobotanical knowledge and indigenous language, folk botanical nomenclature reflects the linguistic rules and cultural phenomena of the local population. Therefore, folk botanical nomenclature is an important resource that enables locals to recognise, remember and use plants, and ultimately to protect plant diversity [3]. Understanding and elucidating folk nomenclature of local plant species is an important part of ethnobotanical and anthropological research [4–7]. Many studies in China have focused on the folk botanical nomenclature of the Dai [2, 8–10] and the Mongolians [11–13]. Some researchers have documented the plant nomenclature of the Yi people in the Daliangshan Yi Autonomous Prefecture in Sichuan Province [3], where the Yi people often use monosyllabic words to name culturally important plants but use Chinese loanwords to name introduced species. The plant-naming system of the Yi people uses binomial and non-binomial forms, and a recent study on plants used in the Bimo religious rituals of the Yi people in Xiaoliangshan [14] found that plants with both binomial and non-binomial names were employed in these rituals. However, this study focused only on the use of plants by the Yi people from the perspective of religious rituals, and it is unclear whether the same nomenclature is employed for plants used for other purposes.

Hengduan Mountains, which are a popular area for studying biodiversity. The combination of the monsoon climate and the complex mountain environment makes it one of the most abundant alpine flora regions in the world [15, 16]. Various ethnic groups who live in this region, including the Yi people, depend on the region's flora for survival: plants are used for medicine, food, feed, fuel, dyes, spices, landscaping, religious ceremonies, and other purposes [17–19].

The Yi nationality is one of the oldest ethnic groups in China, with a population of 9.8 million [20]. The Yi people are widely distributed in Yunnan, Sichuan, and Guizhou provinces in southwestern China [21]. The Yi language belongs to Tibeto-Burman languages, and there are six dialects altogether. Liangshan Yi Autonomous Prefecture in Sichuan province is the main settlement area of the Yi people in China, with about 2.3 million Yi people living here [21]. The religious form of Yi nationality is in the advanced stage of primitive religion. It is a complex religious system with ancestor worship as the core, nature worship, totem worship and polytheistic belief [22]. The Yi people is a mountainous nationality with a livelihood of half farming and half grazing [23]. Due to its complicated historical origin and numerous branches, there are more than one hundred appellations for the Yi nationality. There are different opinions about the origin of Yi nationality in academic circles. Some scholars believe that the Yi nationality originated from the ancient Qiang tribe  in northwest China, which migrated south to the banks of the Jinsha River  and merged with many indigenous tribes [24]. However, after the further study, more scholars believe that: the Yi nationality is an indigenous people in southwest China. They have long been active in the Wumeng Mountain  and Jinsha River basin [25]. The Yi people experienced great migration and gradually divided into six tribes the Wu, Zha, Nuo, Heng, Bu and Mo, all of them gradually settled into the vast areas of southwest China and Southeast Asia [26, 27].

Xiaoliangshan lies in the north-western part of Yunnan Province within the Hengduan Mountains. The Yi people living in Xiaoliangshan progressively migrated there from the Daliangshan and they now constitute the main ethnic group in this area [28, 29]. In the past, the Liangshan Yi people belonged to the Nuo and Heng tribes of the six Yi tribes. They migrated into the Liangshan area along the Jinsha River and became the main ethnic group in the Liangshan area. At present, there are many research on ethnobotany of the Yi nationality in Daliangshan [3, 30]. Academic research on the Xiaoliangshan Yi people has focused primarily on the cultural heritage of the Yi ethnic group from the perspective of anthropology [28, 29, 31], whereas no systematic research has investigated their ethnobotanical knowledge. Combined ethnobotanic and anthropologic studies of the Yi ethnic group would enable the folk botanical nomenclature used by the Yi community in Xiaoliangshan to be established, and such research would contribute to preserving traditional botanical knowledge and promoting and protecting biodiversity within this region.

Therefore, this study aimed to document and analyse the folk botanical nomenclature of the Yi ethnic group in Xiaoliangshan. We aimed to answer the two following questions: (1) What are the rules for the plant nomenclature used by the Yi people in Xiaoliangshan? (2) What are the similarities and differences between the plant folk nomenclature of the Xiaoliangshan Yi people and those of the Yi people in the Daliangshan, who have the same cultural heritage, but live in a different environment? This paper examines the significance of their plant nomenclature methods and the effect that folk botanical nomenclature has on protecting biodiversity and preserving traditional ethnobotanical knowledge.

## Methods

### Study area and introduction to the Yi people

Xiaoliangshan (lat. 26° 36'–27° 56′ N; long. 100° 22′–101° 15′ E) is situated in the northwest of Yunnan Province within the middle section of the Hengduan Mountains. It lies on the border of Sichuan and Yunnan province and has a temperate monsoon climate characterised by warm and moist summers, cold and dry winters, and four distinct seasons [32]. Its primary soil types are subalpine meadow soil, dark brown soil, and subalpine desert soil [33]. Due to its unique geographical location and climatic conditions, there is abundant and diverse flora within the area. According to a former biodiversity inventory of this region, Xiaoliangshan has has 6 vegetational forms, 17 formations, and 1943 species of plants [34].

**Fig. 1:**
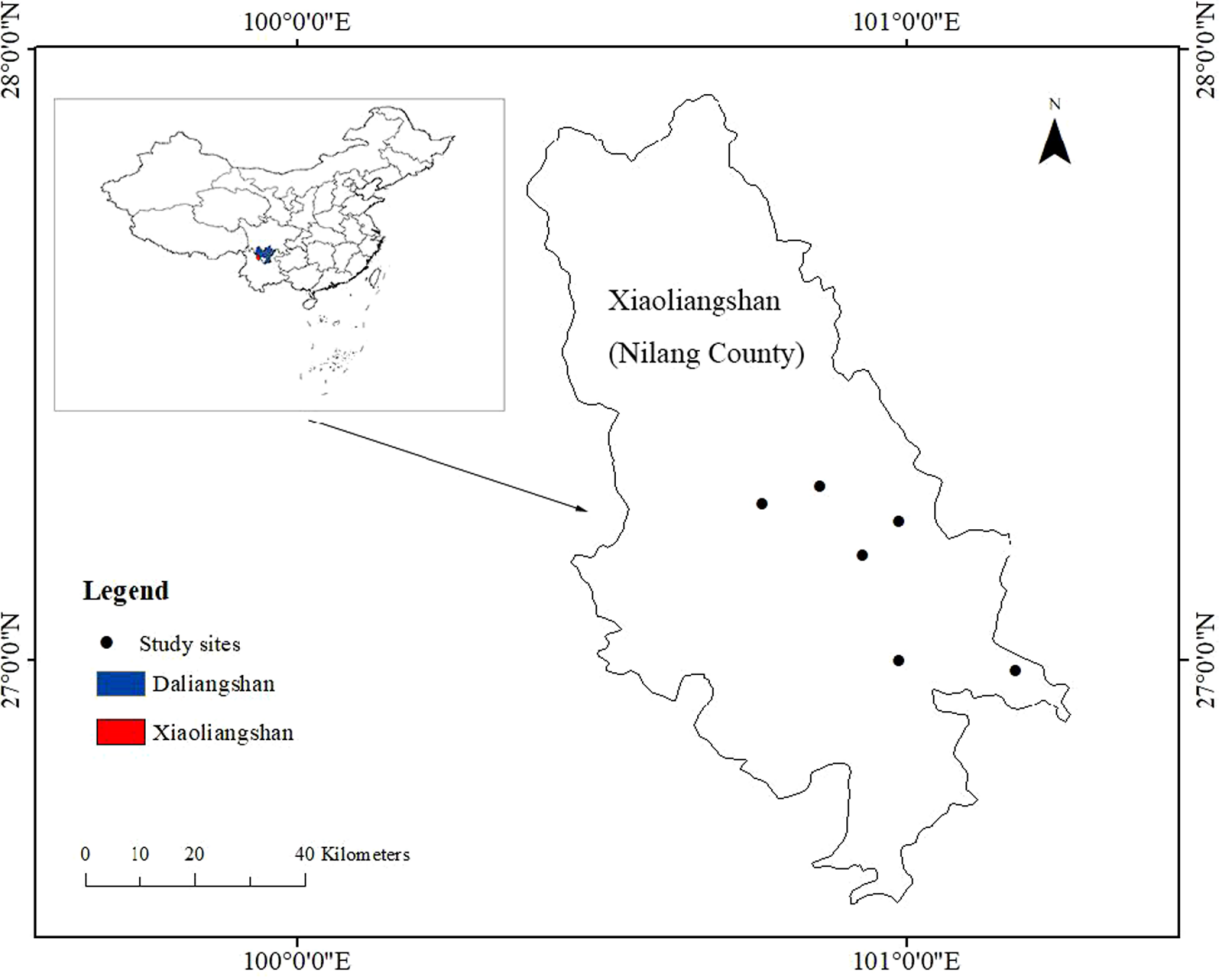
The location of study area: Xiaoliangshan and Daliangshan

Liangshan area is a geographical concept. It is home to the largest group of Yi people in China. The Yi people call the Liangshan area as  , which means the densely forested alpine area. In the present administrative divisions, Liangshan area is divided into Daliangshan and Xiaoliangshan. Daliangshan belongs to China's Sichuan Province including Xichang City, while Xiaoliangshan on the other side belongs to China's Yunnan Province including Ninglang County, Lijiang City. "Da" means "big" and "Xiao" means "small" in Chinese. In fact, people divide Liangshan into "Daliangshan" and "Xiaoliangshan", not only from the perspective of the difference in area size, but also from the residential history of Yi people in the two places by the size of population. In this study, Xiaoliangshan refers specifically to Ninglang Yi Autonomous County of Yunnan Province (Fig. 1).

In this study, we conducted ethnobotanical research in 14 villages and 3 communities within six townships in the eastern part of Xiaoliangshan (Table 1). The Yi people are the main ethnic group within the selected research location, and their traditional lifestyle is well preserved in these communities. According to some studies, the Yi people progressively migrated to Xiaoliangshan from the Daliangshan, and they have eventually become the main ethnic group in this region [28, 31]. In the early nineteenth century, the Yi people in Xiaoliangshan made a living through animal husbandry, farming, and hunting and gathering [35]. Traditional Yi dwellings are made of wood or clay-and-wood [36], and their staple foods include potato, buckwheat, oats, corn, and turnip [37]. Grilling and boiling are commonly used cooking methods [37]. The Yi people firmly believe in animism and worship nature. They also believe that all living things originate from snow, which they consider to be the common ancestor of animals and plants [38]. In the Bimo belief system, the Bimo (a ritual specialist or priest) presides over all major religious activities, including offering prayers and sacrifices [39, 40]. The Yi people in Xiaoliangshan have their own language and script and they use the northern Yi dialect in their daily communication [41].

**Table 1:** Surveyed locations within study area

Town	Village/community	Longitude	Latitude	Altitude(m)	Population
Dàxing town	Well-off homes community	100.861411E	27.304879N	2255	2329
Dàxing town	Riverside Homes Community	100.865977E	27.284771N	2255	2840
Dàxing town	Happy Homes Community	100.864976E	27.306978N	2255	6613
Nínglì township	Nínglì Village	100.765049E	27.251272N	2400	4956
Nínglì township	Báicǎopíng Village	100.71238E	27.174713N	2400	2043
Lànníqìng township	Lànníqìng Village	100.983124E	27.225657N	2850	2891
Lànníqìng township	Dàerdì Village	100.940823E	27.275785N	2750	2398
Xīnyíngpán township	Xīnyíngpán Village	100.926102E	27.172216N	2500	4476
Xīnyíngpán township	Dōng fēng Village	100.919985E	27.187754N	2654	3441
Xīnyíngpán township	Máojiāxiāng Village	100.945282E	27.138304N	2600	4052
Pǎomǎpíng township	Pǎomǎpíng Village	100.987172E	26.996425N	2680	4009
Pǎomǎpíng township	Shālìpíng Village	101.013091E	26.969145N	2720	3297
Pǎomǎpíng township	Yángchǎng Village	101.045571E	26.937666N	2480	1728
Chánzhànhé township	Chánzhànhé Village	101.180402E	26.98326N	2900	4163
Chánzhànhé township	Sāngǔshuǐ Village	101.077553E	26.973122N	2900	1627
Chánzhànhé township	Gànhǎizǐ Village	101.135092E	27.066066N	1680	1387
Chánzhànhé township	Wànmǎchǎng Village	101.095586E	27.033905N	2900	923

### Ethnobotanical survey and data collection

**Fig. 2:**
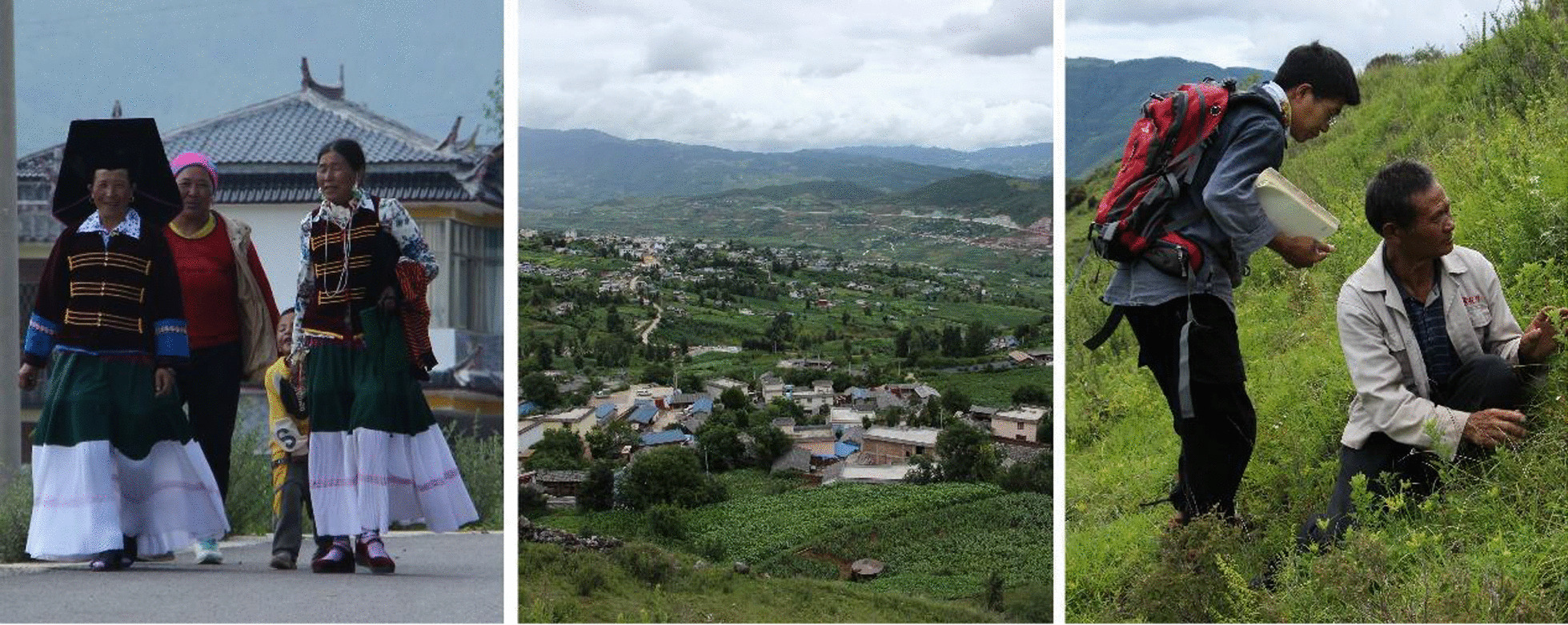
Yi women in traditional dress & Yi village & interviewing in the wild (from left to right)

We conducted several systematic ethnobotanical surveys and investigations in Xialoiangshan from September 2019 to August 2021 (Fig. 2). We used snowball sampling to recruit a total of 266 informants, including 151 males and 115 females. The informants held various occupations, such as local farmers and herdsmen, Bimo practitioners, students, forest rangers, and folk doctors. Key informant interviews and semi-structured interviews were conducted with the informants upon their consent. The interviews were conducted at the informants' homes, fields, shrub, and pine forests, and at sacrificial ritual locations. The first author of this article is a local member of the Yi ethnic group, whose mother tongue is the Yi language. To facilitate communication with the informants and ensure the integrity of the acquired information, all interviews were conducted and documented in Yi language. During each interview, the informants were asked the following pre-prepared questions: (1) What plants do you usually use and how do you use them? (2) What are their names? (3) Can you explain the meaning of their names?

Finally, voucher specimens of the different plants were collected in the nearby fields, farmland, and along roadsides, under the guidance of the key informants. All the collected voucher specimens were authenticated by each member of the research team in charge of this study, based on the publication "Flora of China" [42] and then stored at the Herbarium of the Kunming Institute of Botany, Chinese Academy of Sciences.

### Data analysis

After informant interviews, Microsoft Excel 2016 (Microsoft Corporation, http://www.microsoft.com/) was used to compile the collected data. Acai Yi input (https://www.cr173.com/soft/642454.html) was employed to transcribe the handwritten notes into the corresponding Excel tables. The information collected in the informant interviews served as the basis for our research on the folk botanical nomenclature and classification rules of the Yi people in Xiaoliangshan.

## Results

### Plant species used by the Yi community in Xiaoliangshan

We collected a total of 3088 use reports and extracted 228 folk names of local plants, belonging to 107 families, 178 genera, and 226 species (Table 2). The record of each useful plant includes the following information: plant name in the Yi language and Yi language phonetic name, Latin name, family name of the plant species, voucher specimen number, and the number of use reports.

**Table 2:**
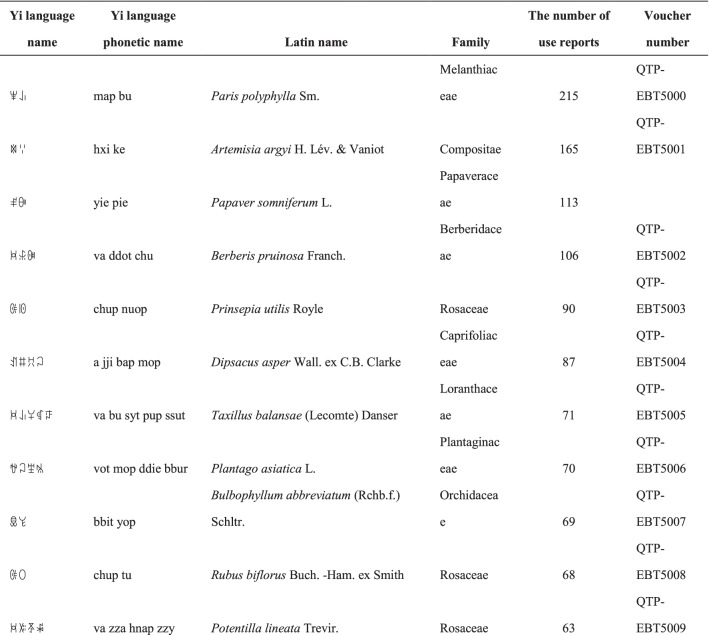

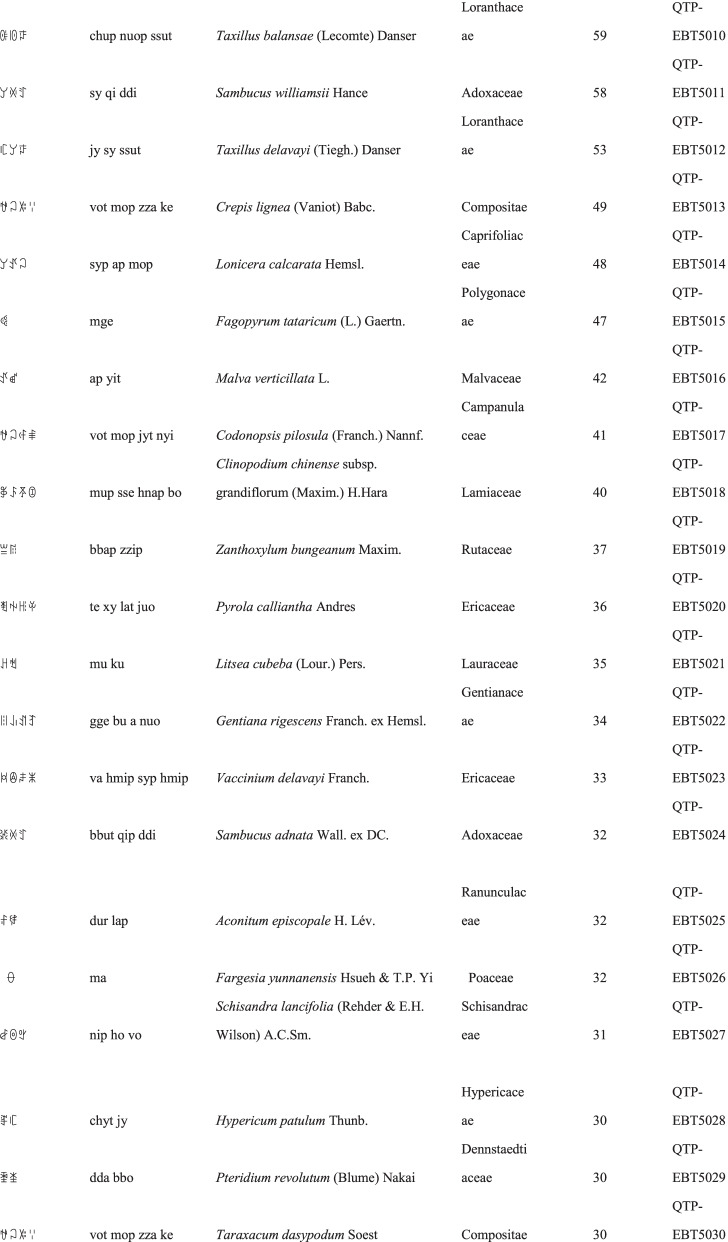

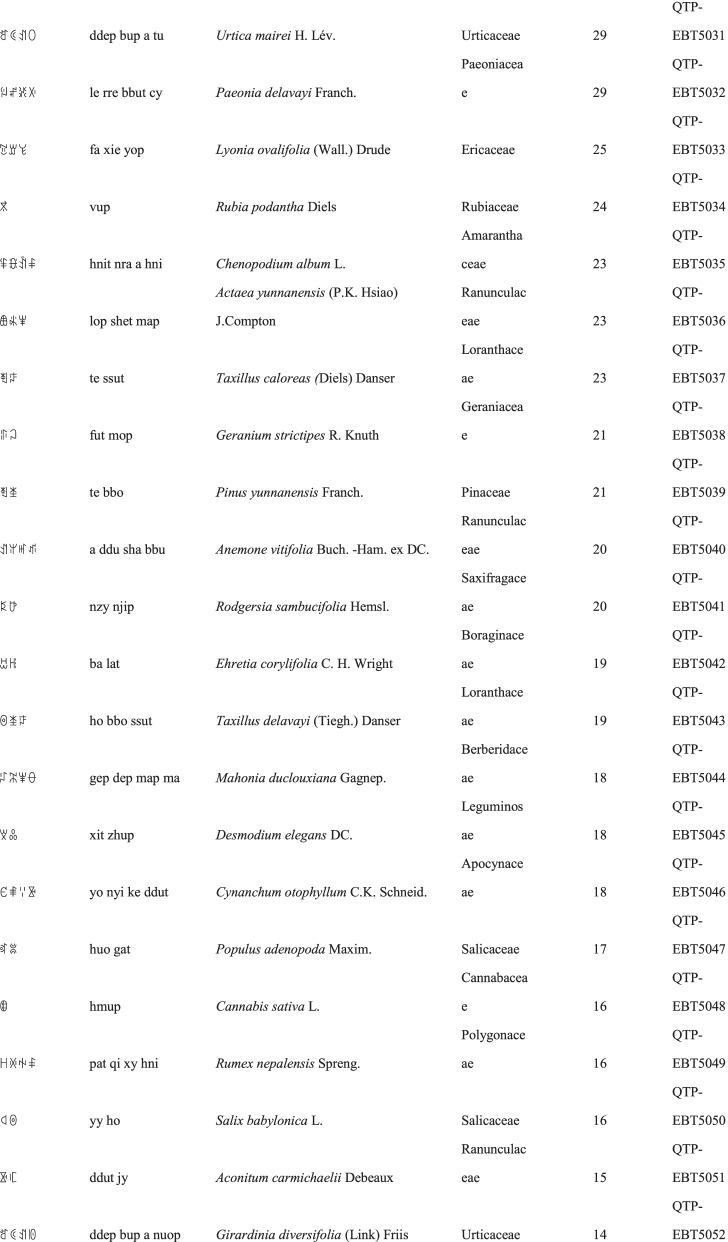

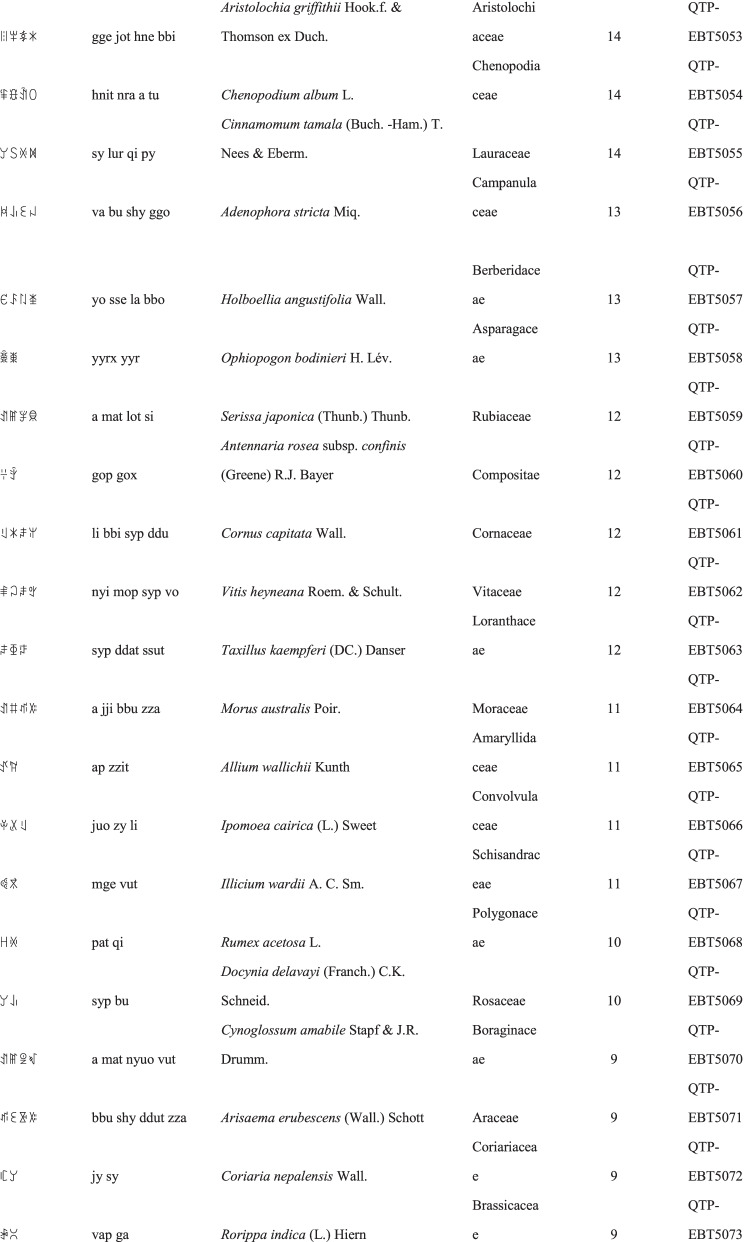

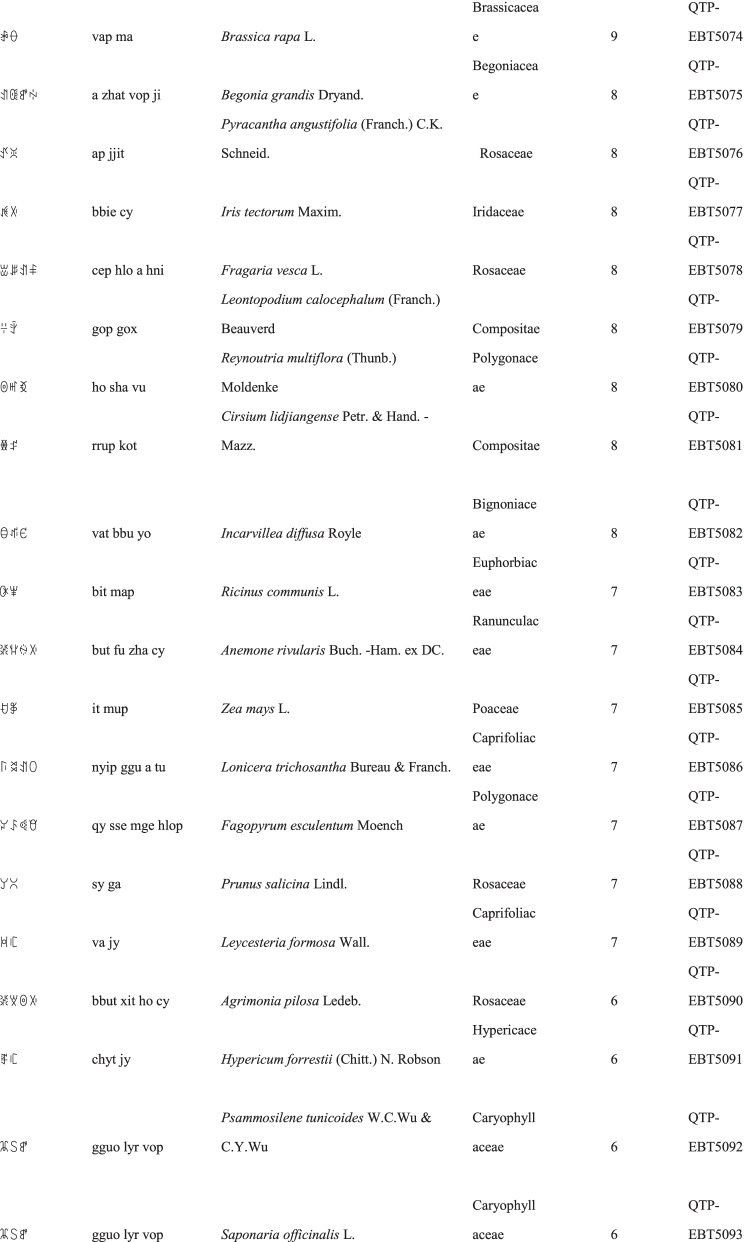

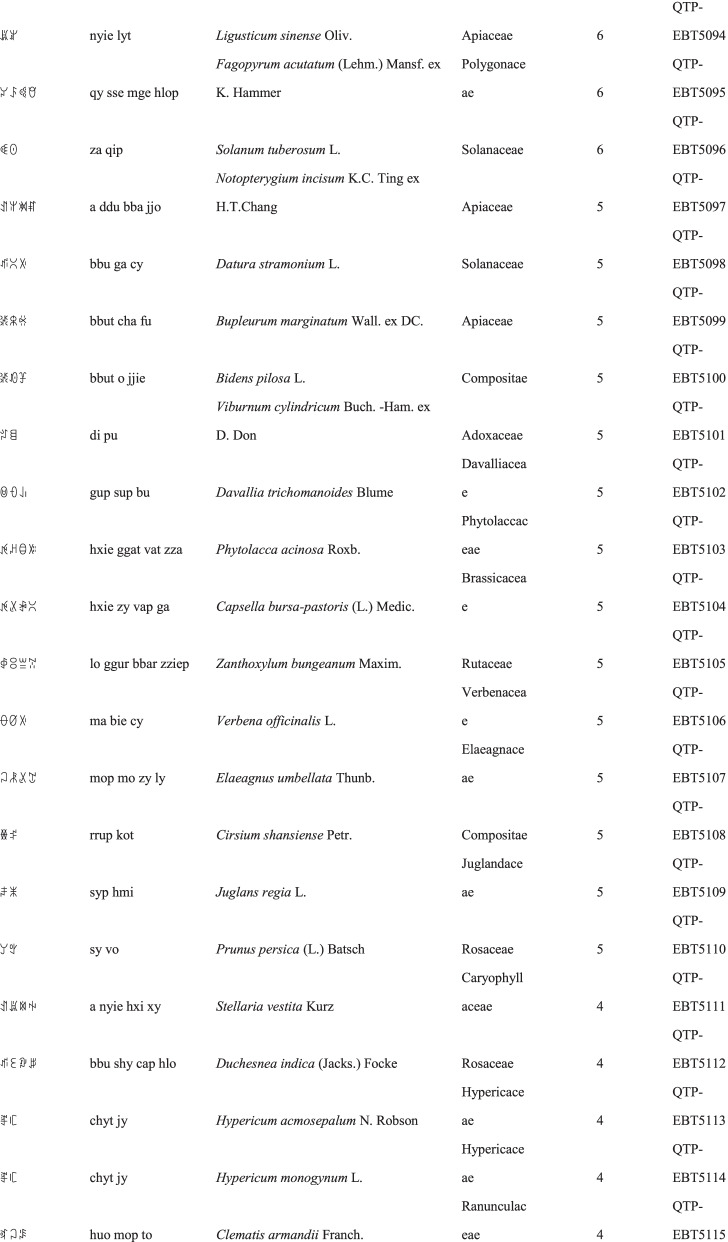

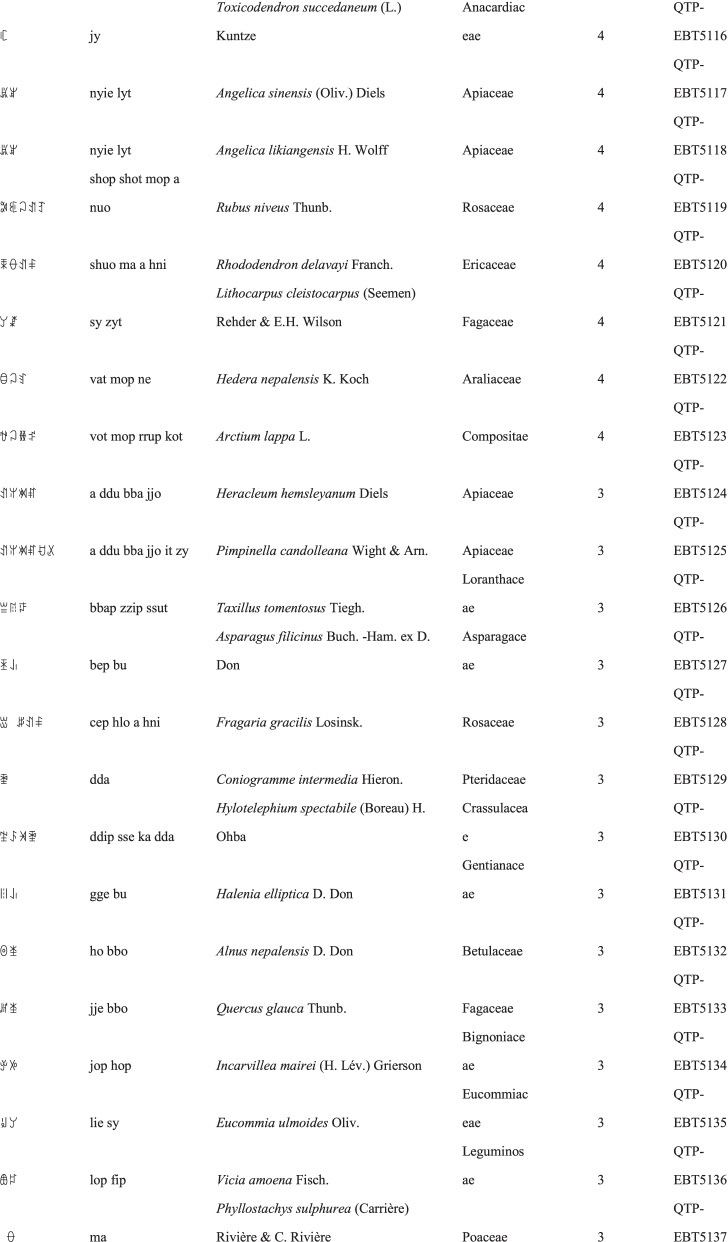

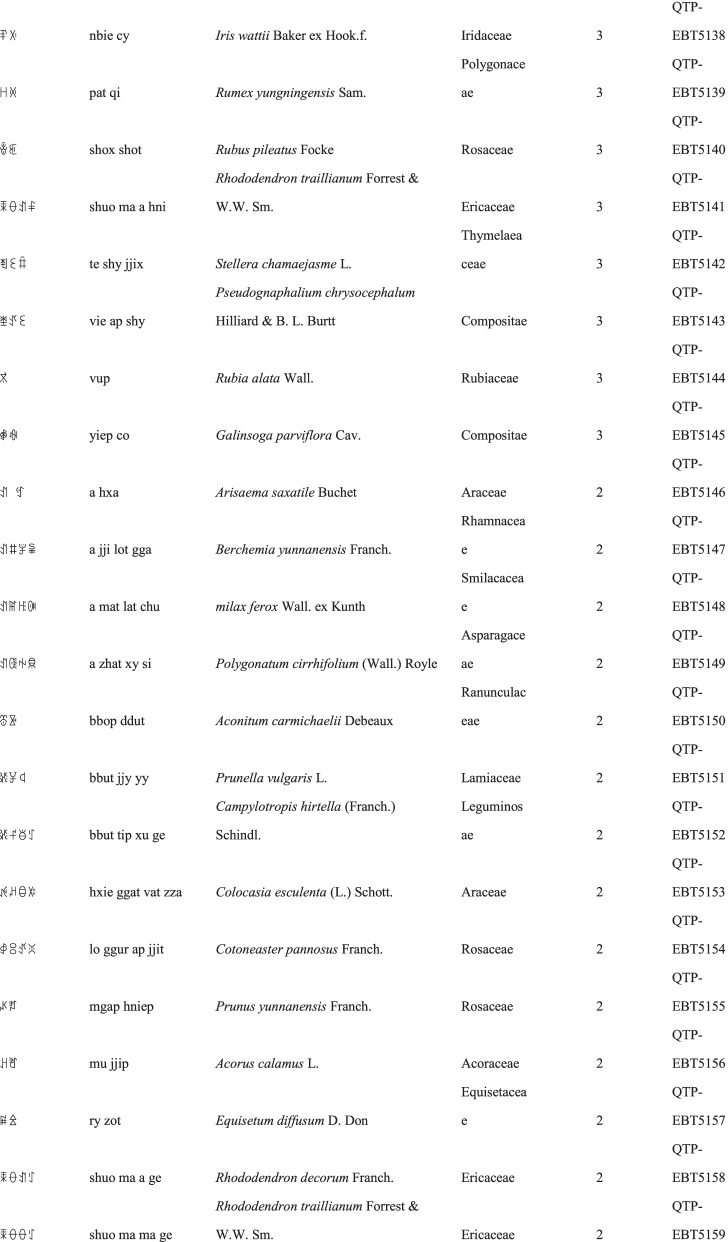

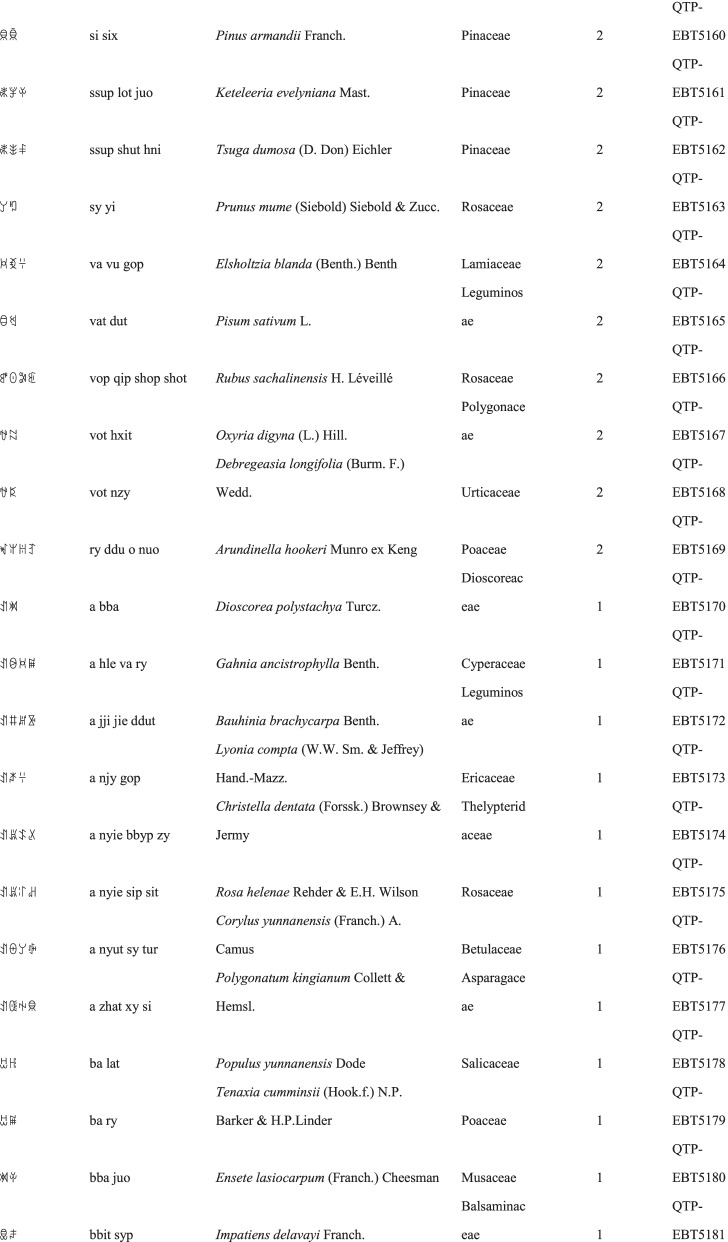

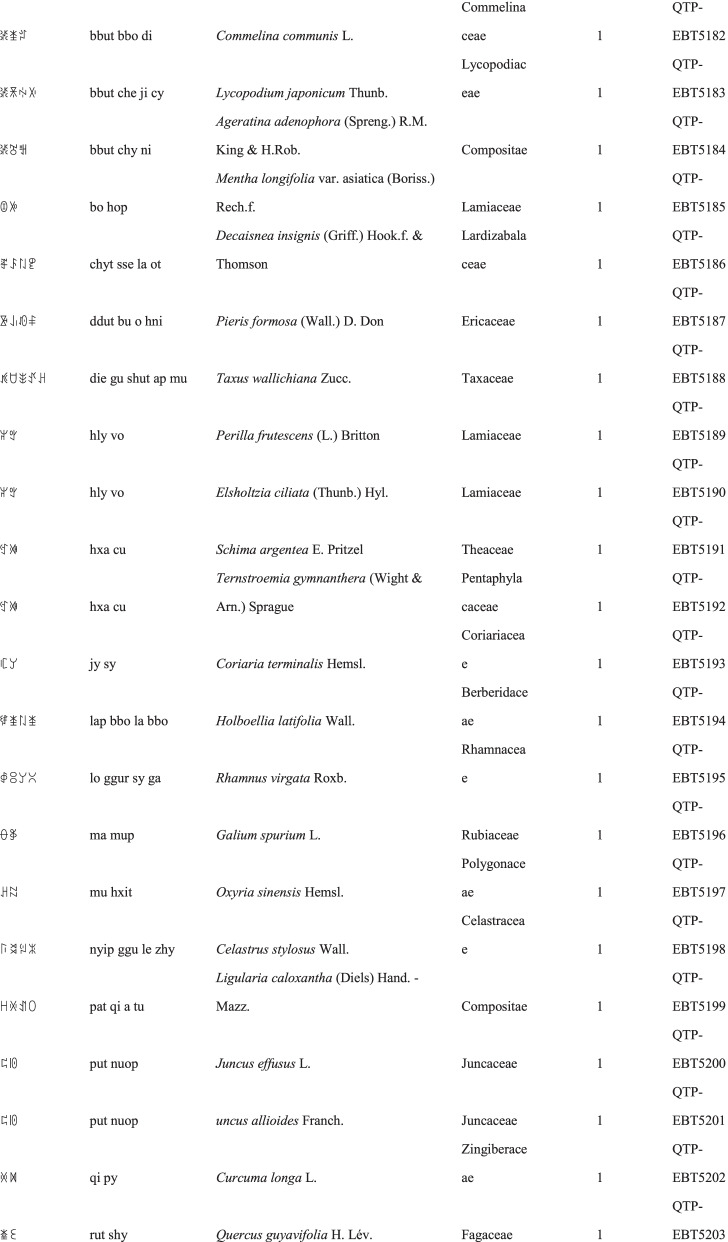

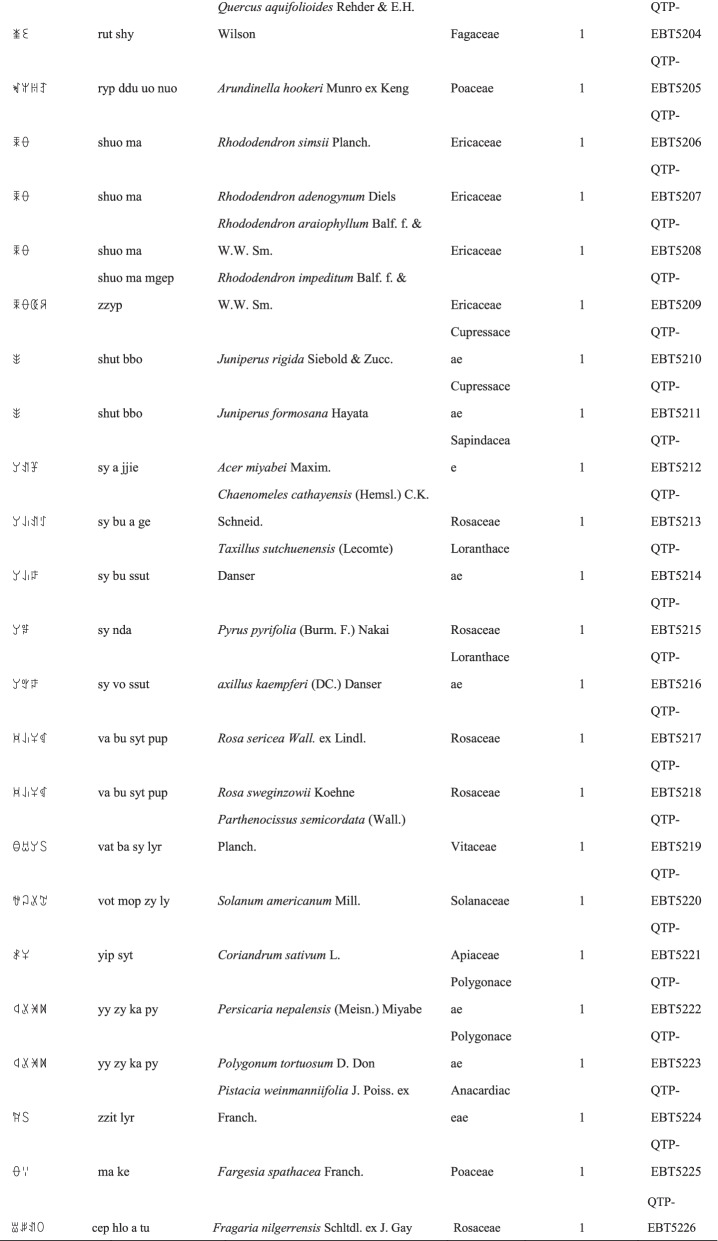
Catalogue of plants used by the Yi people in Xiaoliangshan, Yunnan Province

### Folk nomenclature of plant species in the Xiaoliangshan Yi community

**Fig. 3:**
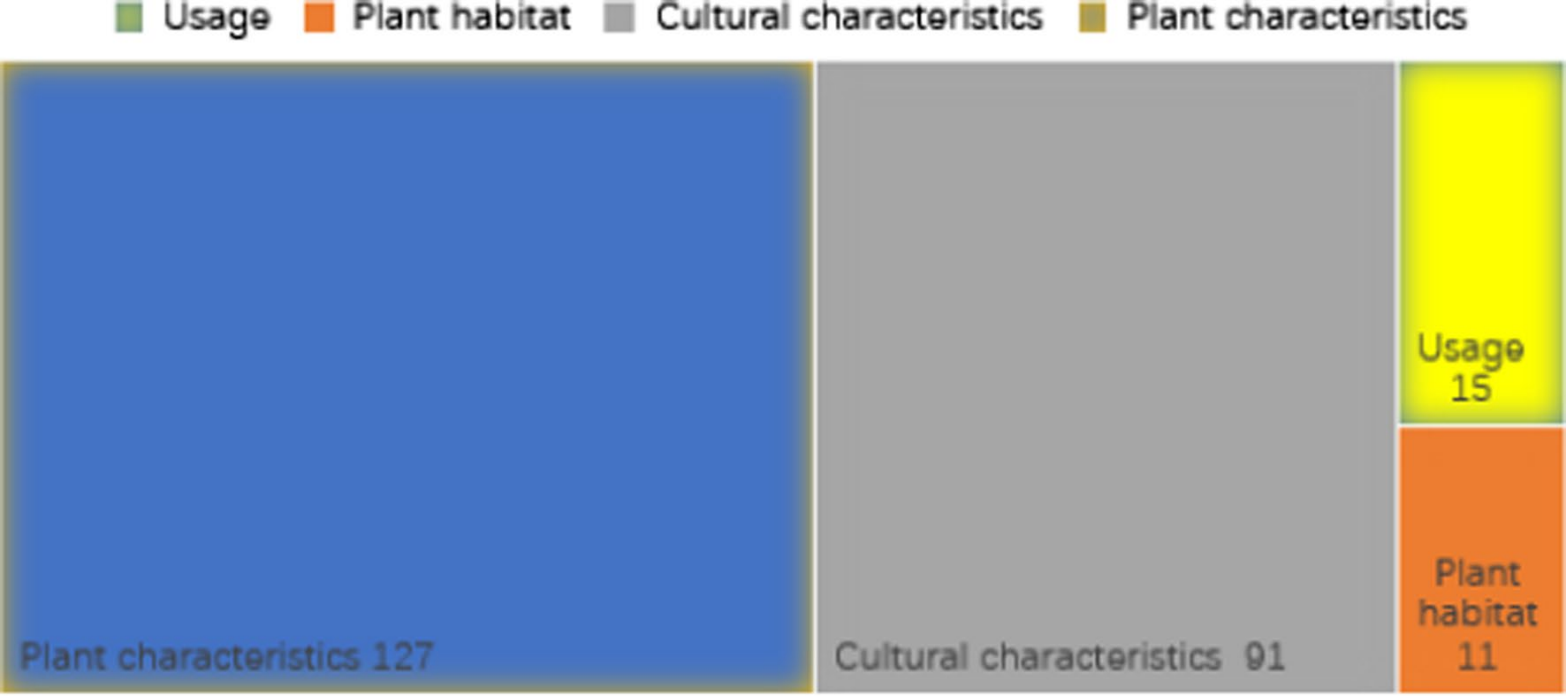
Folk nomenclature criteria for naming plant species in the Yi community of Xiaoliangshan. *Note*: The numbers represent the plant species named using each method

Based on the plant names listed in Table 1, the folk nomenclature criteria for naming local plants used in the Yi ethnic community are based on the following (Fig. 3): plant characteristics (127 species), cultural characteristics (91 species), usage (15 species) and plant habitat (11 species), and these are described in the following sub-sections.

#### Plant names based on characteristics

In this study, we documented 127 species with indigenous names that are based on plant characteristics. These species can be divided into four categories (although some species overlap categories), as follows: plant morphology (two types), plant taste, and plant scent. Of the 127 species, 99 names are based on plant morphology, and these are divided into two types: the first directly reflects the morphological characteristics of the plant and the second uses animal-related concepts and characteristics to describe the plant. In this second nomenclatural group, many of the plants have animal names (Table 3). Examples of plants in these categories are as follows: the locals use the term,  (Yi language phonetic name: *bba jjo*), in the Yi language for plants from the Umbelliferae family, which relates to the hollow stem of these plants; the Yi name for *Bidens pilosa* L. is  (*bbut o jjie*), which means "pitchforked-head grass"; and *Anemone vitifolia* Buch. -Ham. ex DC., which is also known as wild cotton, is named  (*a ddu sha bbu*), which relates the wool-like surface of the plant's achene to the hair of the fox. In addition, the leaf apexes of *Polygonatum kingianum* Collett & Hemsley and *Polygonatum cirrhifolium* (Wall.) Royle, which belong to the Polygonatum genus, are rolled downwards like a bird's claw, and these are named  (*a zhat xy si*), which means "magpie's claws”. Of the 127 plant species with names based on plant characteristics, 26 reflect the colour of the plant; for example, the Yi name for *Pseudognaphalium chrysocephalum* (Franch.) Hilliard & B.L.Burtt is  (*vie ap shy*), which means "yellow flower".

**Table 3:**
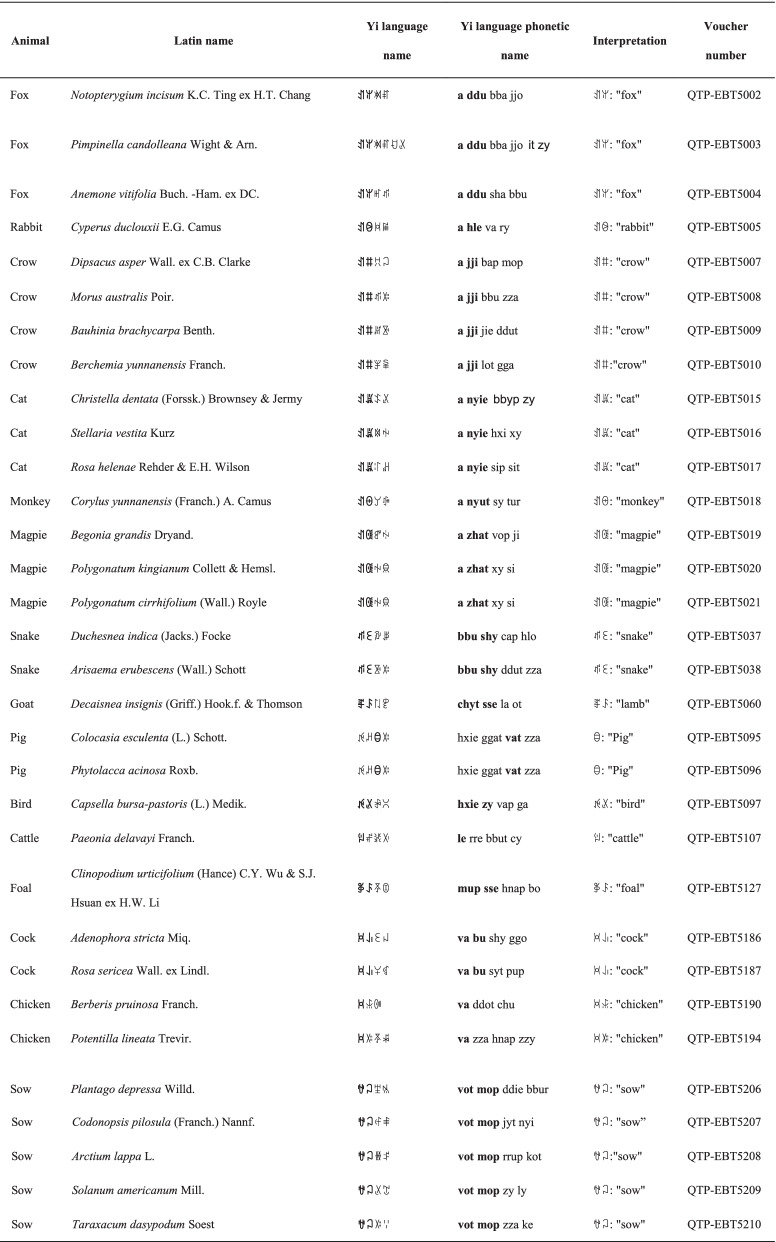
Plant names based on animals in the Yi language

Animal-related words in the Yi language and the Yi language phonetic name are shown in bold

In addition, the names of nine species relate to the plant's taste. For example, *Prunella vulgaris* L. is named  (*bbut jjy yy*), which means "honey grass", and it is named in relation to the honey-like taste of its nectar. Furthermore, the Yi name for *Begonia grandis* Dryand. is  (*a zhat vop ji*), which means "magpie's sauerkraut", and it is so-named because of the sauerkraut-like taste of its stem.

Finally, one plant species is named based on its scent: *Ageratina adenophora* (Spreng.) R.M. King & H. Rob. is named  (*bbut chy ni*), which means "stinky grass", because the whole plant has a distinctly unpleasant odour.

#### Plant names based on habitat

Many plant names in the Yi language are based on their native habitat (Table 4). Terms that describe the plant's habitat (such as the Yi word,  , which means "wild") are often used in the plant’s name. For example, the Yi name for *Cotoneaster pannosus* Franch. is  , which means "firethorn that grows in the wild". This word distinguishes it from *Pyracantha angustifolia* (Franch.) C.K. Schneid., which is commonly planted around local dwellings. The Yi name for *Hedera nepalensis* K.Koch is  , in which  means "cliff" and  means "bead" because this plant is often found on cliff walls and it produces round bead-like fruit. Similarly, the names of many plants that generally grow near water or a swamp have the prefix  or  , which mean "water" and "swamp", respectively; for example, *Persicaria nepalensis* (Meisn.) Miyabe is named  in the Yi language and the willow tree is called  .

**Table 4:**
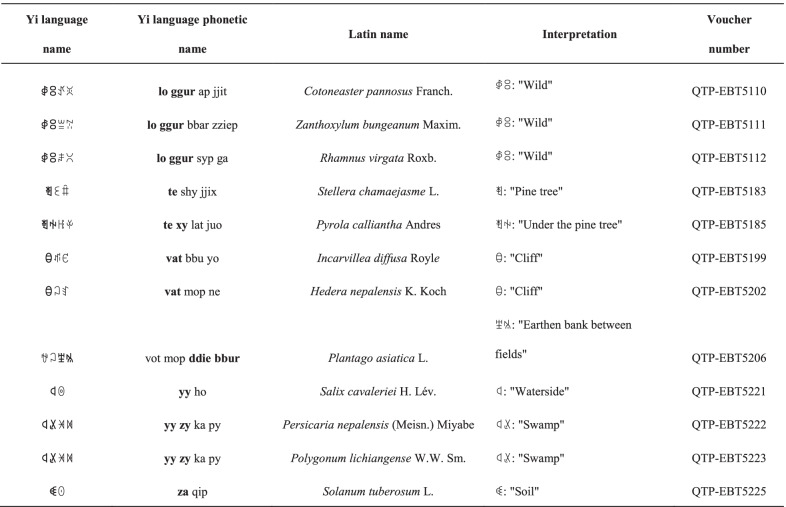
Plant names based on their habitat in the Yi language

Habitat-related words in the Yi language and the Yi language phonetic name are shown in bold in the table

#### Plant names based on culture

Cultural heritage is another important element reflected in the folk botanical nomenclature of the Yi people. The influence of culture on the botanical nomenclature of the Yi community is reflected in the two types of names used: the first type is based on the Yi ethnic culture and the second is based on the combined effect of the Yi and Han cultures. Of the documented plants, the names of 71 species are based on the traditional culture of the Yi people; most of these plant names contain semantically vague phonetic symbols, such as  , which are transmitted orally. There are 18 species of plants with names that reflect the fusion between the traditional Yi culture and the Han culture, and most of these plants are of economic importance (Table 5). Of these, 11 are used for medicinal purposes, six are used as fodder, and one is used as food. Most of these plant names are derived from Chinese transliteration: some are direct transliterations of the Chinese name into the Yi language, and some have a Yi-language prefix added to a Chinese transliteration; for example, the folk name for *Ensete lasiocarpum* (Franch.) Cheesman is  . This Yi name is romanised as "bba juo" which sounds like its Chinese name "ba jiao". Lycopods are called  , which is romanised as "*bbut che ji cy*"; this is a transliteration of the plant's common Chinese name "*chou jin cao*" with the prefix  added to indicate a herbaceous plant.

**Table 5:**
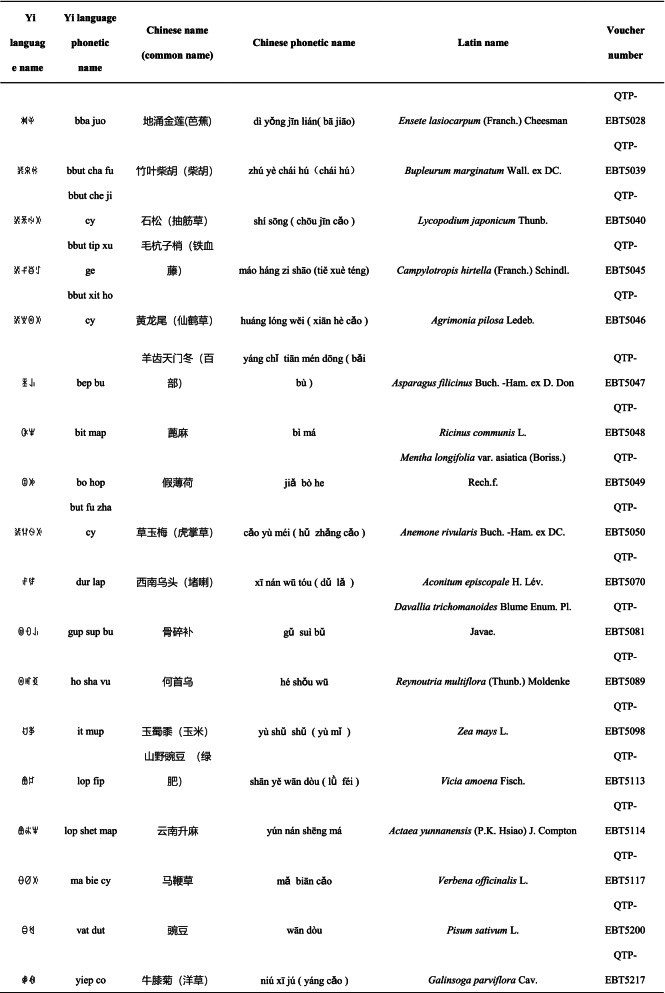
Chinese loanwords in the folk plant names of the Yi community in Xiaoliangshan

#### Plant names based on their common usage

Naming plants based on their common usage is another method of nomenclature used by the Yi people in Xiaoliangshan (Table 6), and of the documented species, the names of 10 plants directly reflect their use. For example, *Paeonia delavayi* Franch., which is commonly used by the locals as strain-injury medication for humans and cattle, is named  , which means "strain injury medicine for cattle". Similarly, *Iris wattii* Baker ex Hook.f. is often used by the locals to treat pneumonia, and its Yi name is  , which means "pneumonia medicine". *Rubus sachalinensis* H. Léveillé is locally called  . When its fruit matures, the locals begin turnip planting. The Yi term  means "planting turnips"; therefore, the plant's name directly reflects its indicator function.

**Table 6:**
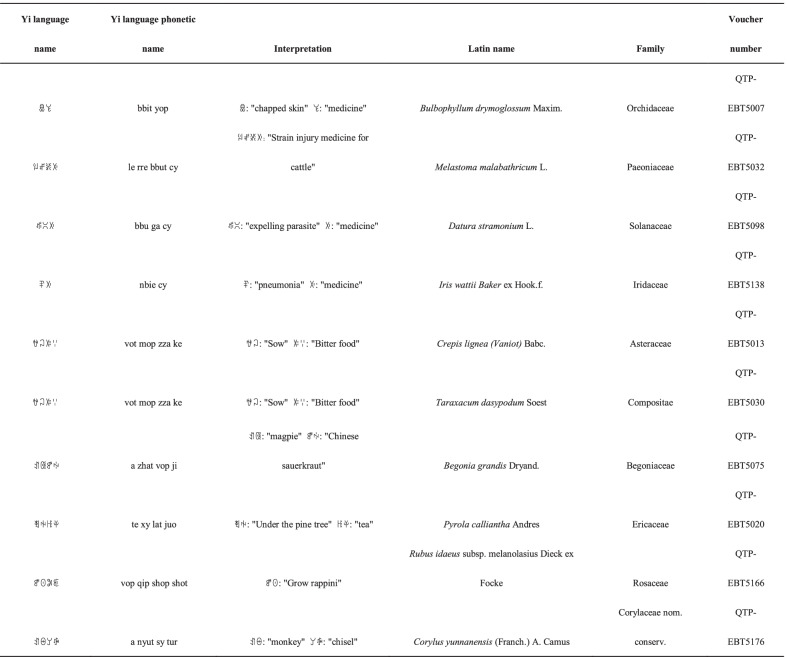
Plant names based on their common use by the Yi community in Xiaoliangshan

### Analysis of the basic structure of traditional plant names of the Yi people in Xiaoliangshan

**Fig. 4:**
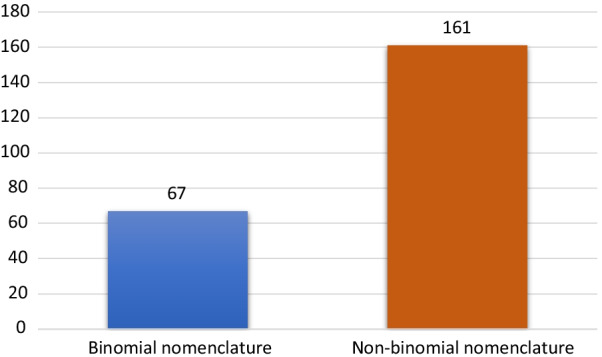
The basic structure of folk plant names used by the Yi people in Xiaoliangshan

In the folk nomenclature system of the Yi people in Xiaoliangshan, plant names have a binomial or non-binomial structure (Fig. 4). A binomial folk plant name consists of two Yi words: one of these is the core or the primary name and the other is a modifier used to describe or clarify the core word. A non-binomial plant name consists of one Yi word. Of the local plants documented in this study, 67 species have binomial names and 161 have non-binomial names. The following examples show the binomial structure of folk botanical names in the Xiaoliangshan ethnic community, where a modifier is added to the core word to highlight its characteristics:

#### *Example 1*

Latin name: *Ageratina adenophora* (Spreng.) R.M. King & H. Rob.

Yi name:  (core word) +  (modifier).

Meaning: smelly (modifier) + herb (core word).

#### *Example 2*

Latin name: *Rhododendron decorum* Franch.

Yi name:  (core word) +  (modifier).

Meaning: Big (modifier) + Azalea (core word).

Plant names with a non-binomial structure consist of one semantically ambiguous core word or a Chinese word transliterated into the Yi language; for example,  (*Zanthoxylum bungeanum* Maxim.),  (*Allium wallichii* Kunth) and  (*Asparagus filicinus* Buch.-Ham. ex D.Don).

### Correspondence between plant names and species in the folk nomenclature of the Yi people in Xiaoliangshan

**Fig. 5:**
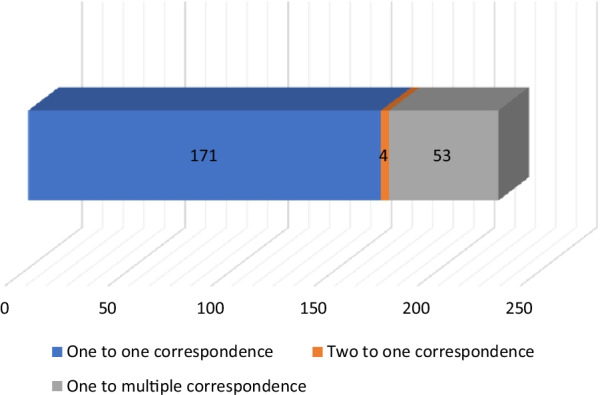
Correspondence between folk botanical names used by the Xiaoliangshan Yi people and plant species. *Note*: The different colours represent the corresponding relationships between the folk names of plants used by the Yi people and plant species: blue indicates a one-name-to-one- species relationship; Orange indicates a two-names-to-one-species relationship and grey indicates a one-name-to-multiple-species relationship

This study found that not all folk plant names and taxonomic species have a one-to-one correspondence; some plant species have multiple folk names, and one folk name may be used for multiple species (Fig. 5). The name to species correspondence is elucidated as follows:

1. One folk plant name corresponds to one species. Of the folk names, 171 correspond to one plant species; for example, the folk name  (*a jji bbu zza*) corresponds only to *Morus australis* Poir.;  (*bbu shy ddut zza*) corresponds only to *Arisaema erubescens* (Wall.) Schott;  (*chup nuop*) corresponds only to *Prinsepia utilis* Royle;  (*dda bbo*) corresponds only to *Pteridium revolutum* (Blume) Nakai;  (*gep dep map ma*) corresponds only to *Mahonia duclouxiana* Gagnep.;  (*huo gat*) corresponds only to *Populus adenopoda* Maxim.;  (*jy bbo*) corresponds only to *Toxicodendron succedaneum* (L.) Kuntze;  (*li bbi syp ddu*) corresponds only to *Cornus capitata* Wall. and  (*mu ku*) corresponds only to *Litsea cubeba* (Lour.) Pers..
2. Two folk names corresponding to one plant species. Of the plant names, four have two folk names corresponding to one scientific name. *Chenopodium album* L. is an edible wild plant commonly used by locals for food and fodder. As the locals classify it as two different plants, it has two different Yi names:  (hnit nra a hni) and  (hnit nra a tu). Similarly, *Rhododendron traillianum* Forrest & W.W. Sm. has two corresponding Yi names,  (shuo ma a hni) and  (shuo ma ma ge).
3. One folk name corresponding to multiple plant species. Of the plant names, 53 have folk plant names that correspond to multiple plant species. For example, four different plant species correspond to the Yi name  (chyt jy): *Hypericum acmosepalum* N. Robson, *Hypericum monogynum* L., *Hypericum forrestii* (Chitt.) N. Robson, and *Hypericum patulum* Thunb.; two different species correspond to the Yi name  (jy sy): *Coriaria nepalensis* Wall.; and two different species correspond to the Yi name  (shut bbo): *Juniperus rigida* Siebold & Zucc. and *Juniperus formosana* Hayata.

### Comparison between folk botanical nomenclature of the Yi people in Xiaoliangshan and the Yi people in the Daliangshan

**Fig. 6:**
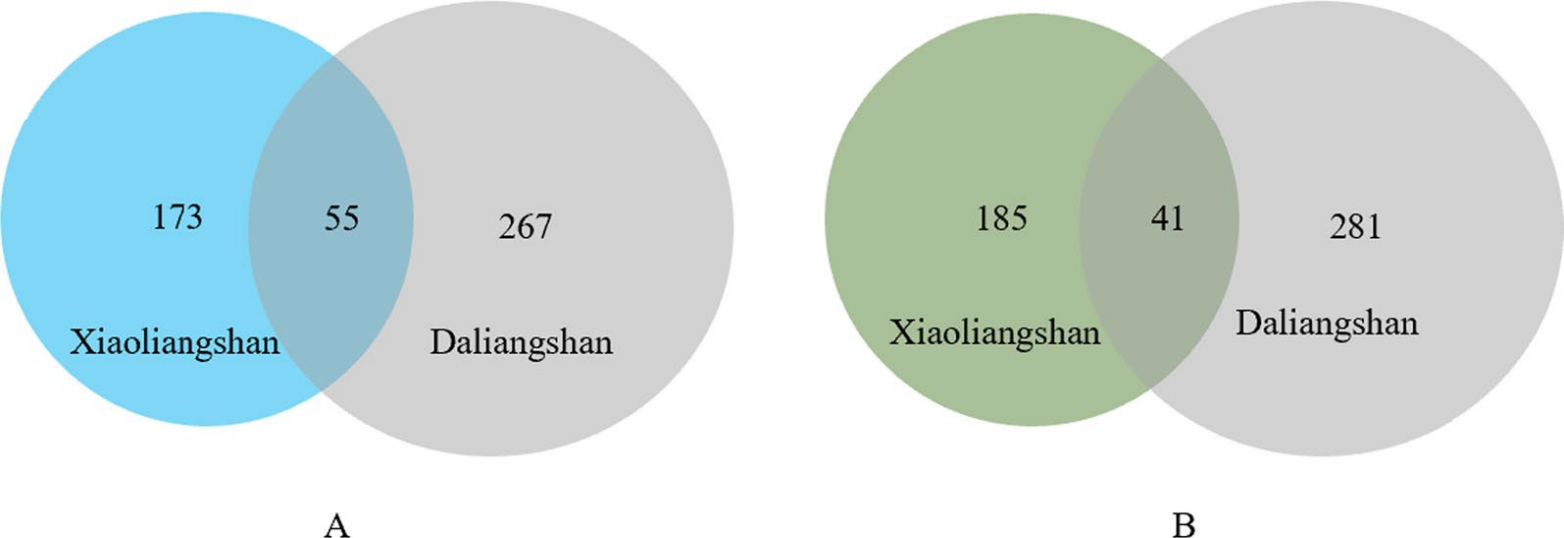
Comparison between the folk botanical nomenclature of the Yi people in Xiaoliangshan and the Yi people in the Daliangshan. *Note*: A shows the overlap between the names of Yi plants in Xiaoliangshan and those in Daliangshan; B shows the plants used in both places and the overlap

We compared the folk botanical nomenclature of the Yi people in the Daliangshan [3] with that of the Yi community in Xiaoliangshan (Fig. 6) and found that the plant names and usages of the Yi people in the two places overlapped to a certain extent. More specifically, the two places have 55 plant names in common (Fig. 6A), corresponding to approximately 24% of the total number of plant names collected in Xiaoliangshan. However, only 18 out of the 55 names represent the same species in both places and the remaining names represent different species. Most of these 18 identical plant species have been used by the local people for a very long time and they have non-binomial Yi names (for example  ). The other 37 plant names that are common to both places refer to different plants; however, the plants belong to the same family or genus in modern taxonomy, or they have some similar attributes. For example, the Yi name  is used for three species of the Pinaceae family: in the Daliangshan it refers to *Abies fabri* (Mast.) Craib and *Larix potaninii* Batalin, whereas in Xiaoliangshan it refers to *Tsuga dumosa* (D. Don) Eichler. In addition, the Yi name  represents three different species of the Artemisia genus: *Artemisia annua* L. and another species of wormwood in the Daliangshan, and *Artemisia argyi* H. Lév. & Vaniot in Xiaoliangshan. In the Daliangshan, the Yi name  represents *Crataegus pinnatifida* Bunge and *Crataegus scabrifolia* (Franch.) Rehder, whereas in Xiaoliangshan, it refers to *Docynia delavayi* (Franch.) C.K. Schneid.. Similarly, in the Daliangshan, the Yi word,  , refers to Populus sp. L., whereas in Xiaoliangshan, it refers to *Ehretia corylifolia* C.H. Wright.

### Analysis of “key plants” in folk botanical nomenclature of the Yi nationality in Xiaoliangshan

Different plants play different role in the daily life of the Yi people in Xiaoliangshan, and their importance is also different. Through interviews, we summarized many important plants in the daily lives of the Yi people in Xiaoliangshan. These plants often have the following characteristics:

(1) Plants with monosyllabic non-binomial structured names. A total of 13 species of plants of this type were recorded. For example,  (*Juniperus rigida* Siebold & Zucc.),  (*Rubia alata* Wall.),  (*Toxicodendron succedaneum* (L.) Kuntze), etc.

(2) Plants with  in the name. In the Yi language,  means "tree". The Yi people in Xiaoliangshan often use the word  as the name suffix for woody plants. In this study, 21 species of plants of this type were recorded. The life forms of these plants are often tall woody plants such as *Cornus capitata* Wall., *Docynia delavayi* (Franch.) C.K. Schneid., *Lithocarpus cleistocarpus* (Seemen) Rehder & E.H. Wilson, etc.

(3) The plants used in Yi nationality’s traditional folk customs including weddings, funerals, sacrifices, the new year of Yi ethnic group, and the torch festivals are also important in Yi nationality’s daily life. This type of traditional folk plant culture is widespread in the life of the Yi people in Xiaoliangshan. A total of 38 species of this type of plants have been recorded in this study. For example, *Fargesia yunnanensis* Hsueh & T.P. Yi, *Pinus yunnanensis* Franch., *Rhododendron decorum* Franch., etc.

The above-mentioned plants are mostly "sacred" plants recognized by the Yi people in Xiaoliangshan as indispensable in daily life of the Yi people. The Yi people also pay special attention to their protection and utilization.

## Discussion

### Characteristics of folk plant nomenclature of the Yi people in Xiaoliangshan

Many ethnic groups name plant species based on what the plant resembles [2, 9, 43, 44]. This method reflects a direct approach of recognising plants through the human senses, and it is based on the plants' visual appearance and taste. All such information is contained in the indigenous plant name. Similarly, the Yi people in Xiaoliangshan named plants based on their characteristics, and the names are often related to the characteristic shape, colour, smell, or taste of the plant. In addition to directly describing plant characteristics, the folk plant names used by the Xiaoliangshan Yi people are often based on animals, a nomenclature practice that is common in other places [3, 45–47]. The frequent use of animal names for plants can be explained by the traditional livelihood of the Yi people, which involves various animals. Some studies have reported that to adapt to the demands of the mountainous environment in southwestern China, the Yi people formed a lifestyle based on farming and pastoral practices, and their dependence on livestock has thus been relatively high [22, 23]. It is therefore likely that when naming local plants, some of the salient features of a plant would stimulate a certain sensory response in the observer and cause them to associate the plant with a familiar object, which was ultimately used in the name selected for the plant. Due to the semi-pastoral lifestyle of the Yi people, it would be easy for an observer to assign suitable animal characteristics to a plant and use them to describe the plant, which is a nomenclature practice similar to that of the Mongolian herders [46].

Of the indigenous plant names of the Yi people in Xiaoliangshan, many include words that describe the plant's habitat, such as water, swamp, and field. This practice is also common in the folk plant nomenclature of the Mongolian and the Tung ethnic groups [48, 49]. Adding a habitat-related word to the plant's name would likely help distinguish it from other species and make it easier to find and collect. For example, the Yi people believe that *Rhamnus virgata* Roxb. is a wild plum (*Prunus salicina* Lindl.), so they use a habitat-based name to reflect the similarities and the differences between the two plant species. Another example is *Plantago major* L., which is a type of plant often used by the locals as pig feed; the Yi name of this plant reflects its habitat, which provides a clue to locals about where to find it.

Folk plant nomenclature is also based on oral traditions [50–52]. This study found that many plant names that are transmitted orally often contain semantically ambiguous phonetic symbols, and this finding is consistent with those of other studies of folk plant names used in traditional rituals within this area [14]. It is believed that in this type of nomenclature, in which the plant name is assigned directly and passed on orally, the unique name refers to the biological organism itself, and no further semantic analysis is therefore required.

In addition to the local Yi culture, the Han culture has also impacted the folk nomenclature of the Yi community in Xiaoliangshan. Many foreign plants have been introduced to the community, and the majority are used for medicinal purposes. The Yi people either directly transliterate the Chinese names of the introduced plants or add a Yi-language prefix to the Chinese transliteration. It is believed that these Chinese loanwords were introduced to Xiaoliangshan in a brief window of time during the 1960s when there was a lack of medical care in China, and the "barefoot doctor" policy was implemented [53]. The local government conducted basic medical training for barefoot doctors lasting 4 to 6 months [54], and they were later employed in local villages as healthcare providers, which may have helped spread knowledge about Chinese herbal medicine in the Xiaoliangshan area. The increased use of borrowed Chinese names may also be related to the popularisation of standard Mandarin in basic education, ethnic integration, and the transformation of traditional lifestyles in the Xiaoliangshan region, and this was determined by another study based on the folk botanical nomenclature of the Yi people in Daliangshan [3].

With respect to the function-based plant nomenclature of the Yi people in Xiaoliangshan, the indigenous names employed reflect the plant’s use or its value to humans and animals. This is similar to the function-based plant names used by the Han ethnic group [24]. For example, the Yi name of *Paeonia delavayi* Franch., which is used by the locals to treat injuries in humans and cattle, directly reflects the plant’s use. It is believed that this practice is also related to the traditional livelihood of the Yi people. The Yi people are nomadic farmers, and cattle are the main source of power used in their traditional farming practices [25]. As wasteland reclamation is labour intensive, both humans and animals, but especially cattle, would often suffer strain injuries. Therefore, the plant that was used as a folk remedy for strain injuries has been given an indigenous name that reflects this use. Similarly, the Yi name for *Rubus sachalinensis* Lévl. reflects its indicator plant function. The Yi people in Xiaoliangshan have a long-standing practice of turnip cultivation, and this overwintering vegetable is sown seasonally and continues to be a staple food of the Yi people [26]. However, turnips are formed approximately three months after flowering [55, 56], and such a short growth cycle means that locals need to correctly assess the optimum sowing time for the crop. The fruiting period of *Rubus sachalinensis* Lévl. is from August to September [57], which coincides with the time when the locals begin turnip planting. Therefore, the indigenous name for *Rubus sachalinensis* Lévl. reflects this indicator plant’s function of notifying the Yi people that it is time to sow turnips.

This study found that the Yi people named useful plants using a binomial and non-binomial structure. This is consistent with the findings of a study focusing on plants used in religious rituals [14]. The binomial structure for the botanical nomenclature used by the Yi people is similar to that of the Dai and Han ethnic groups [9, 58]. It is believed that this naming structure is used due to practical considerations: it enables the locals to learn important information about different plants, including their life form, habitat, and functions, which ultimately makes it easier to recognise and remember useful plants. The non-binomial names tend to reflect the characteristics of the local language; these names are generally transmitted orally using semantically ambiguous phonetic symbols. Plants such as *Fagopyrum tataricum* (L.) Gaertn., *Cannabis sativa* L., and *Oryza sativa* L. have been cultivated by the Yi people for a very long time [59–61], and the ancient Yi names of these plants have a monosyllabic no-binomial structure. They are often used as root words when naming more complex plants, which indicates their important roles in the lives of the local Yi people [62].

This study found that there were three types of correspondence between plant names used by the Yi people in Xiaoliangshan and the plant species, namely: one plant name for one plant species, two plant names for one plant species, and one plant name for multiple plant species. These correspondence types are similar to those found by Raven et al., who studied the folk nomenclature and taxonomy of indigenous plants in Mexico [4], and to those of the Chinese Mongolian ethnic group [46]. Investigating the correspondence between folk plant names and plant species enables us to better understand how the Yi people in Xiaoliangshan perceive and recognise plants. This is especially true when multiple indigenous names are given to one plant species, or when one indigenous name corresponds to multiple plant species. For example, the locals classify *Chenopodium album* L. as two plants, which is reflected by the folk nomenclature. Both names emphasise colour  , even though *Chenopodium album* L. is a plant that is widely distributed and has many morphological variations [63]. Another example is that four different species of Hypericum are all named  in the Yi language. These species are primarily found in southwestern China [57], and they are all important medicinal plants used in Xiaoliangshan to treat the same health problem. It is thus believed that they share one indigenous name in Xiaoliangshan because they have a similar form and function.

### The influence of national cultural similarities and differences on plant naming

Cultural differences are an important factor that underlies various people’s conventions for plant naming. For example: (1) Differences in languages of different nationalities will lead to differences in plant naming. In this study, the Xiaoliangshan Yi people have many proper nouns for plant names, most of which are phonetic shells with no specific meaning, which are also common in the folk plant names of other ethnic groups [12].

This proper noun inherited by members of the cultural group representing the biological organism itself. The proper noun itself has no specific meaning. It belongs to the cultural characteristics of a specific nationality. (2) The differences in the use of plants by different ethnic groups lead to differences in plant naming. For the same plant, local people with different cultural backgrounds use plants differently. Consequently, leading to differences when naming such plants. For example, in Xiaoliangshan, the root decoction of *Malva verticillata* L. can be used as a medicine for oxytocin, but the Yi name  is a noun passed by word of mouth and has no specific meaning. Therefore, the meaning of this proper noun is not related to the function of the plant. In contrast, the Mongolians named it "taur nogo", which means "Peach vegetable"[12]. The name comes from the fact that the tender leaves of this plant are often eaten as vegetables by Mongolians. In addition, traditional cultures such as different religious beliefs and livelihoods may affect people's naming of plants. The traditional culture of Yi people’s religious beliefs means of livelihood and language deeply influence the naming of plants by Yi people. It is mainly reflected in the worship thought contained in plant names, many animal names, and a wealth of proper nouns.

However, for the same cultural groups living in different geographic environments.

The factors affecting plant naming may not only be caused by cultural characteristics. Ethnobotanists Cassandra L. Quave and Andrea Pieroni stated that regardless of the living environment, the decisions and behaviour of an ethnic group of people are influenced by their culture [64]. Therefore, analyzing the folk plant names of the same cultural group living in different environments can reveal the influence of external factors other than culture on plant naming. The Yi people in Xiaoliangshan and those in the Daliangshan belong to the same ethnic group, but their living environments differ. In this study, the plants referred to by similar plant names in two places were analyzed. The reason for this result may not only be related to culture, because the cultural origin of the Yi people in the two places is the same. This also explains why there are many the same words in the names of plants in these two places.

The Yi people of Xiaoliangshan immigrated from Daliangshan about 200 years ago [28, 29, 31]. Elderly people of Yi nationality in Xiaoliangshan will trace their family tree back to Daliangshan, and some families of Yi people in Xiaoliangshan still maintain marriage relations with Yi people in Daliangshan. Therefore, in this large-scale family migration and intermarriage, the Xiaoliangshan Yi people retain many of the original living habits of their parents. In the end, this traditional plant name was passed down through generations. However, the differences in geographical environment and the influence of other cultures may also cause some changes in plant names by their exploitation of the local flora for living.

### The relationship between folk nomenclature of plant species in Yi communities and biodiversity conservation

Hengduan Mountains is a global diversity hotspot [65]. But accelerated urbanisation progress has resulted in a severe loss of biodiversity within this region [66]. To protect biodiversity more effectively in ethnic minority areas, it is necessary to first preserve cultural diversity, and particularly to protect aspects of ethnic cultures that are closely related to biodiversity. The folk nomenclature of fauna and flora are important parts of cultural diversity and are essential for use in biodiversity conservation [67]. This is reflected primarily in the following two aspects: first, from a local perspective, folk nomenclature reflects an indigenous knowledge and understanding of individual plants and their unique characteristics, and it contains important information about plant attributes. The traditional knowledge constituted by these individual plants, including diverse germplasm and traditional medicine resources that have been used for centuries by the ethnic group, is a treasure trove of material and cultural wealth [68]. Therefore, as an important part of ethnic and cultural diversity, folk botanical nomenclature is extremely relevant in biodiversity conservation practices [69]. Second, from the overall perspective of biodiversity conservation, ethnobiological nomenclature reflects the relationship between living organisms and habitats. It is the indigenous epistemology of a complex natural system involving individual organisms and the environment. The use and knowledge of the folk nomenclature of living organisms permit people with non-scientific backgrounds to participate in biodiversity conservation efforts [70]. Many studies have investigated the relationship between cultural diversity and biodiversity, and the positive effect of regional traditional cultures on biodiversity conservation has been widely recognised in the scientific community [71]. For example, studies have shown that biodiversity and cultural diversity overlap in their geographical distribution [72, 73].

For the Yi people in Xiaoliangshan, folk botanical nomenclature is a rich cultural tradition that was formed as a means of managing and using local plant resources. This traditional knowledge is essential for the protection and sustainable development of local biodiversity.

First, the Yi people often use monosyllable names with non-binomial structures to name plants that are essential in their daily lives. Moreover, the Yi people often worship and protect plants with such names. For example,  (bamboo) is often used to make ancestral spirit bamboo cards in the life of Yi people in Xiaoliangshan.  is the physical substance worshipped by the ancestors of the Yi people in Xiaoliangshan, and it is often given a sacred meaning. For example, bamboo is worshipped in daily life and cannot be destroyed at will.  (pine trees) and  (fir trees) are also very important plants in the life of the Yi people. The Yi people often live at high mountains with lush fir trees when choosing residential areas. They often gather on the edge of fir forests and regard the dense fir forests as a place where gods live. If people break into the fir forest at will and disturb the gods, they will be punished by the gods. Therefore, fir represents the homeland of the gods believed by the Yi people and has a sacred meaning. Interestingly, when an old man from the Yi ethnic group in Xiaoliangshan said that he was about to die, he would say: "I am waiting for a tree", which means "I am a dying person, and I just want to find a tree to cremate myself". The Yi people often choose fir trees and pine trees for cremation. Thse plants with monosyllable names are generally sacred in the life of the Yi people in Xiaoliangshan and cannot be destroyed.

Second, the plant names of the Yi people in Xiaoliangshan also directly reflect the worship of plants. The Yi people in Xiaoliangshan oftenly believe that many plants have the attributes of "god" and are gifts given to patients by "god". If someone collects such plants as commodities for sale, or collects too much, the collector will be punished by the “god”. The typical characteristics of these plants are the names that often have "deterrence", such as  (*Lonicera calcarata* Hemsl.),  (*Taxus wallichiana* Zucc.) and  (*Ophiopogon bodinieri* H.Lév.). Their meanings are "The Queen of the Tree", "The Alpine Tree King", and "The Spiritual Grass" respectively.

In addition, the Xiaoliangshan Yi people usually protect and reasonably use some plants with  in their names. Such plants are usually tall trees, these plants are easy to distinguish in the folk botanical nomenclature. The main source of fuel needed by the Yi people in Xiaoliangshan is firewood, and every household has a firepit. The daily cooking, sacrifices, weddings, and other important activities of the Yi people all revolve around the fire pond. The fuelwood is an indispensable and important source of fuel supply for firepits. Therefore, the Yi people often collect plants such as  (*Lithocarpus cleistocarpus* (Seemen) Rehder & E.H. Wilson) and  (*Pinus yunnanensis* Franch.) as fuelwood.

When collecting firewood, the Yi people collect the branches of plants and will not cut down the entire tree under normal circumstances. However, during cremation ceremonies, building houses, etc., they will have to cut down the entire tree. At this time, the Yi people usually take off some branches of the felled trees and graft them on the stakes of the felled trees. In addition, they will use soil and moss to cover the "wounds" of the stumps.

In general, the folk botanical nomenclature of the Yi people in Xiaoliangshan contains an appreciation of nature and plant biodiversity, which greatly promotes the local Yi people's awareness of the rational use and protection of biodiversity.

From the perspective of cultural heritage, the folk botanical nomenclature of the Yi people in Xiaoliangshan is an integral part of their traditional knowledge, and it needs to be preserved for future generations. In recent years, accelerated urbanisation and the introduction of foreign culture have greatly affected the traditional knowledge of the Yi people in Xiaoliangshan. One manifestation of this trend is the increasing economic migration of young people to large cities [74] and their gradual assimilation into urban society; they thus have fewer opportunities to use their native Yi language. Due to the assimilation process between the Yi people and the Chinese culture, the language is being increasingly affected. In addition, young people from the Yi ethnic group remaining in Xiaoliangshan now use many Chinese loanwords due to the internet and other mass media usage. Certain popular internet terms have already become an integral part of their language on a large scale, and these are gradually replacing the Yi language [75]. Furthermore, under the recent Poverty Alleviation Resettlement policy, many Yi ethnic group members have been relocated from the mountains to urban areas [76]. The most significant consequence of these above factors is the loss of the local language, and language is the core of culture and the means of transmitting traditional knowledge.

The indigenous nomenclature of plant species is a proper naming system that reflects the rules of the local Yi language. Some studies have shown that the loss of native languages in indigenous communities impairs the transfer of traditional knowledge between different generations, lowers their sense of ethnic identity, and adversely affects the mental and physical health of the indigenous people [77]. In Xiaoliangshan, the loss of the traditional Yi knowledge is obvious; for example, during the interviews conducted in this study, we found that the names of many wild plants commonly collected during the Great Famine in China in the 1960s [78] are now only known by a few aged community members. In addition, the names of plants that are still commonly used for medicinal purposes or as feed are only known by middle-aged and older community members. When shown photographs of different plants, the younger community members recognised the plants, but either could not name them in the Yi language or they only knew the names used by the Han ethnic group, even though their parents were very familiar with and used these plants.

This gradual loss of ethnobotanical names equates to a loss of traditional knowledge and ethnic culture. Studies have shown that the potential for humans to acquire resources from nature through language will become increasingly difficult with the loss of languages. Because indigenous languages are closely related to the pharmaceutical knowledge of ethnic groups, it is believed that the demise of indigenous languages will have a greater impact on pharmaceutical knowledge than on the loss of biodiversity [79]. The use of folk botanical names enables us to harness benefits from natural plant resources. Therefore, from the perspective of cultural heritage, creating standardised records of the ethnobotanical nomenclature of the Yi people in Xiaoliangshan and the rules they used to name plants is critical for preserving this valuable traditional knowledge.

## Conclusions

This study used ethnobotany research methods to document the indigenous plant nomenclature of 226 locally used plant species belonging to 178 genera and 107 families. The folk names of plants and their corresponding scientific names have the following three types of relationships: one plant name for one plant species, two plant names for one plant species, and one plant name for multiple plant species. The nomenclature used by the Yi people in Xiaoliangshan has either a binomial or non-binomial name structure, and four primary factors are used to name plant species: plant characteristics, plant habitat, plant-use, and cultural attributes. Among them, cultural characteristics are important factors that determine differences in plant naming. The Yi people in Xiaoliangshan usually use monosyllable non-binomial structure names to name the most important plants in their daily lives. At the same time, the plants with  in the name and "divine attributes" must be rationally used in the daily life of the Yi people and cannot be destroyed arbitrarily. This study of the folk botanical nomenclature of the Yi ethnic group in Xiaoliangshan will help promote the preservation of traditional knowledge and biodiversity conservation in this area. However, this study only focused on an analysis of ethnobotanical nomenclature, and further research is thus needed to determine whether similar nomenclature rules are used for other living organisms, such as animals and fungi.

## Data Availability

Please contact author for data requests.
